# Gold-Coated Temperature Optical Fiber Sensor Based on a Mach–Zehnder Interferometer for Photovoltaic Monitoring

**DOI:** 10.3390/ma18081818

**Published:** 2025-04-16

**Authors:** Bartlomiej Guzowski, Mateusz Lakomski, Krzysztof Peczek, Lukasz Ruta, Maciej Sibinski

**Affiliations:** 1 Department of Semiconductor and Optoelectronic Devices, Lodz University of Technology, 93-590 Lodz, Poland; mateusz.lakomski@p.lodz.pl (M.L.); krzysztof.peczek@p.lodz.pl (K.P.); lukasz.ruta@p.lodz.pl (L.R.); maciej.sibinski@p.lodz.pl (M.S.); 2 Department of Material and Environmental Technology, Tallinn University of Technology, Ehitajate tee 5, 19086 Tallinn, Estonia

**Keywords:** tapered optical fiber, Mach–Zehnder interferometer, temperature, sensor, gold, surface plasmon resonance, photovoltaic system, monitoring

## Abstract

The development of a Mach–Zehnder interferometer based on tapered optical fiber for temperature sensing applications is presented. Two tapers, 24 mm apart, were fabricated on SMF-28e+ using the fusion splicer. The optical structures were coated with a 100 nm layer of gold. The influence of the gold deposition on the temperature sensitivity of the fabricated sensors is presented. The sensor was characterized in O-, S-, C-, and L-bands in a temperature range of 0–70 °C. The highest temperature sensitivity of 72 pm/°C with *R*^2^ = 0.9974 was obtained for the gold-coated sensor. During the investigation, the average transmission loss was low and did not exceed 7 dB.

## 1. Introduction

Over the past few decades, optical fiber sensors have been intensively developed for a great number of applications. Optical fiber sensors are capable of measuring strain [1,2,3,4], pressure [5], temperature [6,7,8], displacement [9,10], and bio-photonic and medical tests [11,12,13]. In Korec et al. [6], the tapered optical fibers surrounded by the cladding formed by a nematic liquid crystal were used as temperature sensors. In Leal-Junior and Blanc [7], three channel thermometers based on FBG are proposed, and in Hu et al. [8], a cascaded optical fiber device of long-period grating and photonic crystal fiber is proposed to measure the temperature. These sensors attracted significant attention due to their compact size and lightweight design, efficiency, robust electromagnetic interference immunity, and long-distance monitoring capabilities. Various designs of optical fiber-based temperature sensors have been proposed, such as distributed optical fiber sensors, which are based on Rayleigh, Brillouin, or Raman scattering [14,15], fiber Bragg grating (FBG) [16,17], long-period gratings [8], tapered optical fibers (TOF) [7], and modal interferometers.

Temperature monitoring of photovoltaic (PV) systems is very important since the efficiency and performance of the PV module are strongly affected by the solar irradiance and its temperature. It is imperative to monitor temperature in PV installations, as excessive heat has the potential to reduce efficiency and shorten the lifespan of solar panels. Overheating can induce thermal stress, which can result in damage to electrical components and wiring. The presence of faulty or damaged elements, such as defective connectors or overheated inverters, can create hotspots that increase the risk of fire. Electrical arcing or insulation breakdown due to excessive temperatures can ignite nearby materials, posing a serious safety hazard. Consequently, the implementation of continuous temperature monitoring is imperative to detect potential failures in a timely manner, thereby mitigating the risk of fire hazards and ensuring the safety of the system as a whole.

Different approaches to temperature monitoring systems are used, such as electric methods with, e.g., thermocouples or Pt100 sensors [18,19] or thermal imaging [20,21]. Recently, temperature sensors based on optical fiber have also been widely investigated by researchers. The sensors based on FBG [22,23] or distributed temperature monitoring systems based on the Raman or Brillouin effect are proposed [24]. The FBG-based temperature sensor for monitoring large-scale PV farms is proposed by Dhanalakshmi et al. [22], and it offers a sensitivity of 6 pm/°C. Another FBG application in PV monitoring was proposed by Nivelle et al. [25], where absolute temperature accuracy was ±0.3 °C. In Santolin et al. [26], the authors confirmed that FBG sensors can be used in PV applications and compared the results with those of traditional PT100 sensors.

Among optical fiber-based methods, the interferometric methods can be utilized in order to measure temperature with an optical fiber. Four categories of optical fiber interferometers can be distinguished: the Fabry–Perot interferometer [27], the Mach–Zehnder interferometer (MZI) [28], the Michelson interferometer [29], and the Sagnac interferometer [30]. Among these, the MZI [31,32] and the MI have gained particular popularity due to their compact structure and advanced sensing capabilities, which can be further enhanced by incorporating a tapered region in the optical fiber [33,34,35].

In a previous study [36], we presented a high-sensitivity temperature sensor based on periodically tapered optical fiber with six and eight tapered regions. However, the higher number of tapered regions makes such a construction harder to fabricate and causes high attenuation in transmission. Therefore, in this study, we propose an optimized version of the temperature sensor with only two tapered regions. However, in order to improve the sensor sensitivity, the surface plasmon resonance (SPR) technique is employed.

In this paper, the experimental investigation of the temperature sensing ability of developed MZI structures is presented. The proposed temperature sensor was characterized in the O-band (1260–1380 nm), as well as in the S-band (1460–1530 nm), C-band (1530–1565 nm) and L-band (1565–1625 nm). In the further part of the article, the sum of these three bands will be referred to as the S+C+L-bands. The obtained results demonstrated that the proposed MZI-based sensor achieved a temperature sensitivity of 72 pm/°C, which is approximately seven times higher than standard FBG gratings.

## 2. Materials and Methods

### 2.1. Sensor Principle

**Figure 1:**
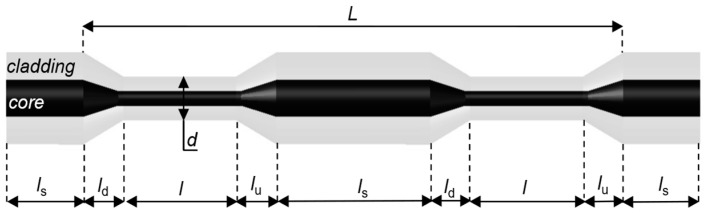
Conceptual construction of periodical tapered optical fiber.

In the context of tapered optical fiber, four distinct regions can be identified, as illustrated in Figure 1. The tapered-down and tapered-up regions are designated as *l*_d_ and *l*_u_, respectively. The waist region, characterized by a length *l*, is positioned between the *l*_d_ and *l*_u_ regions. The fourth element of the TOF is a segment of non-tapered fiber, with its length designated as *l*_s_. The geometry of the sensor, particularly the symmetry and the uniform waist region diameter *d*, are critical parameters in the context of TOF. In order to fabricate the tapered regions, an arc-discharge method can be used [36,37] since this method provides high control, a short-time fabrication process, and is relatively low-cost [38].

The light behavior through the TOF is easy to understand. Due to the abrupt core reduction in a taper-down region, some of the light transferred through the core leaks into the cladding. Subsequently, in the non-tapered fiber section between the tapers, the core and cladding modes travel until they arrive at the second taper. Another similar taper structure placed a few centimeters after the first one enables cladding mode energy to be coupled back from the cladding to the core. Since the core and cladding have different refractive indices, the modes that recombine at the second tapered region are not in phase. The phase difference between the cladding and the core modes, designated as ∆*ϕ*, results in the formation of a Mach–Zehnder interferometer within the sensor [39]. When the phase difference satisfies the condition *φ* = (2k + 1)π transmission dips would appear, where k—is an integer. This phenomenon has been documented in [40,41]. Equation (1) describes the relative phase difference between these interfering modes.

(1) $$∆\phi =\frac{2\pi }{\lambda }\Delta n_{\mathrm{eff}}L$$

where ∆*n*_eff_ is the difference between core and cladding effective refractive indices, *L* the interferometric length, and *λ* the central wavelength of the light.

The intensity at the TOF output is wavelength-dependent and can be expressed as follows (Equation (2)):

(2) $$I=I_{\mathrm{co}}+I_{\mathrm{cl}}+2\sqrt{I_{\mathrm{co}}+I_{\mathrm{cl}}}\cos\phi$$

where *I*_co_, *I*_cl_ are the intensities of the core and cladding modes, respectively.

The free spectral range (FSR) for the transmission is given by Equation (3) [42]

(3) $$\Delta \lambda_{m}\approx \frac{\lambda^{2}}{\Delta n_{\mathrm{eff}}L}$$

(4) $$\frac{d\lambda }{dT}≅\frac{\frac{2L}{2k+1}\left(\frac{\partial ∆n_{\mathrm{eff}\;}dn_{\mathrm{co}}}{dT\;\partial n_{\mathrm{co}}}+\frac{\partial ∆n_{eff\;}dn_{\mathrm{cl}}}{dT\;\partial n_{\mathrm{cl}}}\right)+\frac{\lambda }{L}\;\frac{dL}{dT}}{1-\frac{2L}{2k+1}\;\frac{\partial n_{\mathrm{eff}\;}}{\partial \lambda }}$$

Interferometric sensors which are highly sensitive to the change of the surrounding environment should have a large value of the wavelength shift [43]. This requires the terms of difference of phase between interfering modes and refractive index difference ∆*n*_eff_ between the materials where the fundamental mode and the high-order mode are propagating. Moreover, a high temperature sensitive layer deposited on the tapered optical fiber will influence the thermo-optical properties of the fiber by modifying heat conduction, thermal expansion and refractive index. The thermo-optic effect, in combination with the variation of *L* due to glass thermal expansion, leads to a temperature-dependent modification of the refractive index of the core and cladding [44]. The temperature sensitivity of the sensor can be described by Equation (4) [45].

**Figure 2:**
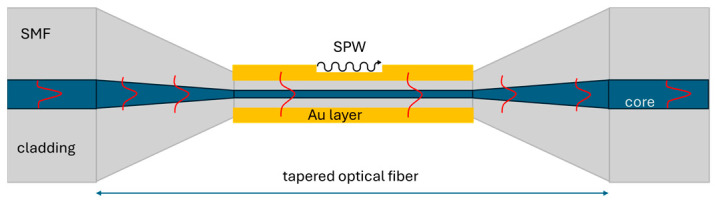
Generation of surface plasmon wave in gold coated taper optical fiber.

In order to improve the sensitivity of the sensor, the newly developed SPR was employed [46,47]. This technique, among the local surface plasmon resonance effects [48], can be characterized by its exceptional sensitivity. It is predicated on the interaction of light with free electrons at the interface between a metal and a dielectric, as shown in Figure 2, resulting in the resonant energy transfer between the light guided within the core and the evanescent wave [49]. Temperature changes cause alterations in the refractive index (RI) of materials in proximity to the metal surface. These RI changes affect the SPR resonance conditions, causing a shift in the resonance wavelength. A variety of optical fiber structures have been investigated by researchers, e.g., D-shape fibers [47], multi-hole fibers [50], S-shape fibers [51], or nano-modified fiber core [52] in order to increase the leakage of evanescent waves. Additionally, tapered optical fibers have been investigated [53] since they enhance the penetration of the evanescent wave, facilitating the generation of surface plasmon polaritons. The smaller the taper diameter, the easier it is for the evanescent wave to reach the metal surface, generating an SP wave and increasing sensor sensitivity. Noble metals, such as gold (Au) and silver (Ag), are most commonly used for coating due to their low chemical reactivity and SPR properties [54,55]. Typically, 30–60 nm thick Au layer [46,56,57,58] and waves from a range of 500 to 800 nm are used to achieve surface plasmon resonance. However, these wavelengths have high attenuation coefficients of 5–10 dB/km, which makes them less useful in wide-area and long-distance sensing applications. For such applications, typical telecom wavelengths (1200–1600 nm) are more suitable. However, to obtain SPR for such bandwidth in a gold layer, the thickness must be increased to the range of 100–200 nm [59,60,61]. Therefore, a 100 nm Au layer was deposited on both waist regions, as shown in Figure 2.

### 2.2. Sensor Fabrication

**Figure 3:**
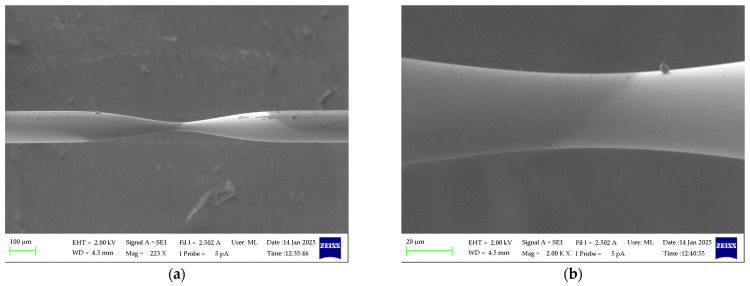
The geometry of TOF developed with *P*_arc_ = 20 mW and *t*_arc_ = 1.6 s (**a**); close-up of a waist region (**b**).

The sensors were fabricated using an arc-discharge method, which is widely regarded as one of the most effective techniques in this field due to its high control, short fabrication process, and relatively low cost. During this research, the Furukawa Fitel S153A fusion splicer was utilized. The MZI sensors consisted of two tapered regions separated by a distance of 2.4 cm. A series of experiments were conducted to investigate the effects of varying arc power *P*_arc_ and arc duration *t*_arc_. The results of these experiments are detailed in Table 1. This method enabled the fabrication of a diverse array of tapered regions. The geometry of the TOF was characterized using a scanning electron microscope (SEM) Zeiss EVO MA10, and the results are presented in Figure 3. The fiber taper looks relatively symmetrical and appears to have a smooth, continuous profile without abrupt changes in thickness. This indicates that the heating and stretching process was well controlled and precisely performed. The image shows no obvious microcracks or roughness that could suggest unevenness in the process. High-quality taper requires precise control of the temperature and forces acting on the fiber. A smooth taper minimizes propagation losses and Rayleigh scattering, which is important for sensing applications. The transmissions of developed sensors were investigated, and for further tests, we decided to use MZI sensors fabricated with *P*_arc_ = 20 mW and *t*_arc_ = 1.6 s since the lowest waist diameter enhanced the interferometry, and the obtained results were the most promising one.

**Figure 4:**
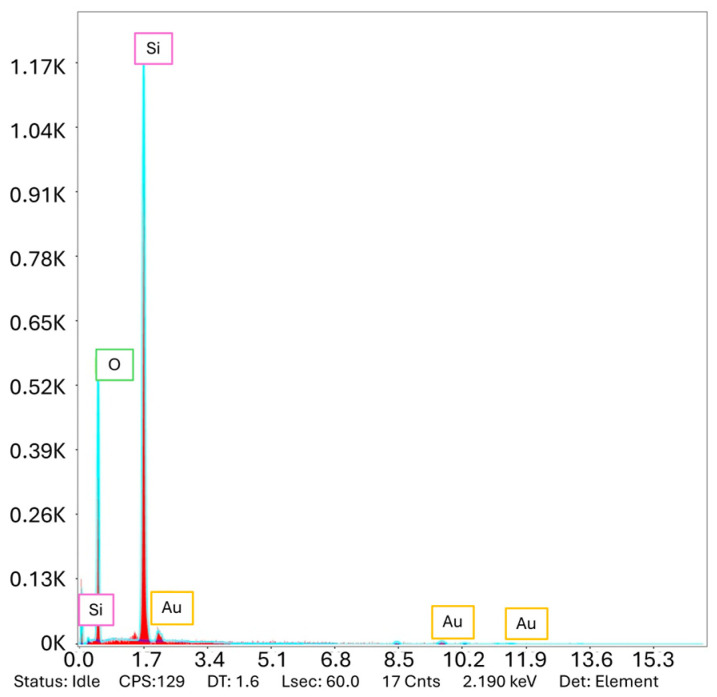
EDS Measurement analysis.

In the subsequent phase of the procedure, the MZI sensors were coated with a 100 nm layer of Au employing the evaporator Balzers BAK550. The thickness and the roughness of the layer were checked with a Dektak 3 surface profilometer and were equal to 105 nm and 4.3 ± 1.4 nm, respectively. The energy dispersive X-ray spectroscopy (EDS) was used to determine the chemical composition of the MZI structures. The spectrum peaks presented in Figure 4, which are characteristic of silicon, oxygen, and gold, provide evidence of the presence of a thin gold layer on the optical fiber.

### 2.3. Experiment Setup and Method

**Figure 5:**
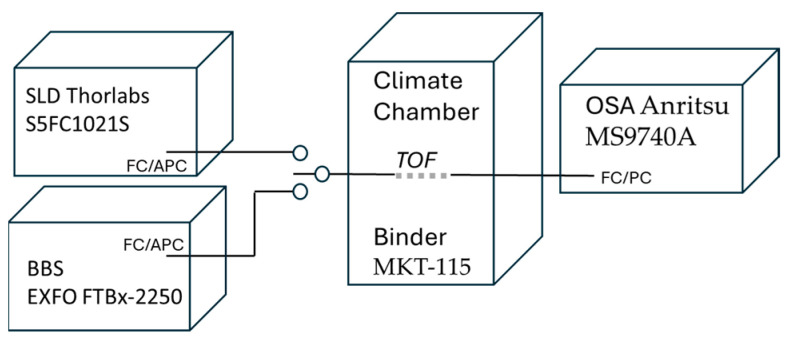
Measurement setup schematic.

During the proposed sensor characterization, the measurement setup depicted in Figure 5 was utilized. The fabricated samples were placed in the climate chamber Binder MKT-115. On the one side, the TOF was connected to either the broadband light source EXFO FTBx-2250 with a range of 1460–1625 nm or Thorlabs S5FC1021S with a center wavelength equal to 1310 nm and 85 nm optical bandwidth. On the other side, the sensor output was connected to an optical spectrum analyzer, Anritsu MS9740A. Based on the measurement setup, the wavelength accuracy readings were equal to ±300 pm for the whole tested spectral range, while the temperature chamber fluctuation did not exceed 0.6 °C. During the research, a single-mode fiber SMF-28e+ from Corning was utilized.

The transmission was obtained by subtracting the spectrum of the light captured by OSA at the output of the optical fiber with tapers from the light source’s spectrum. The sensor was characterized for the heating and cooling processes. The temperature was varied from 0 °C to 70 °C and back to 0 °C, with a 10 ± 0.6 °C step increment.

## 3. Results

### 3.1. Bare Tapered Optical Fiber

**Figure 6:**
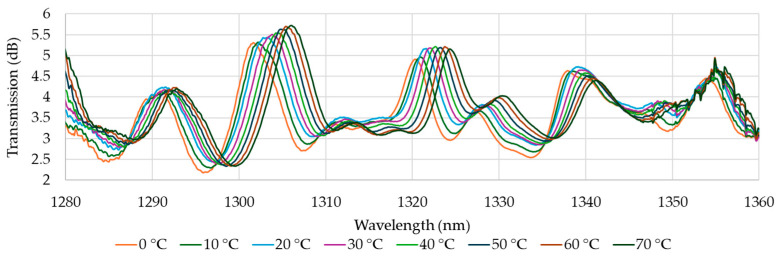
The transmission characteristics in O-band as a function of temperature.

**Figure 7:**
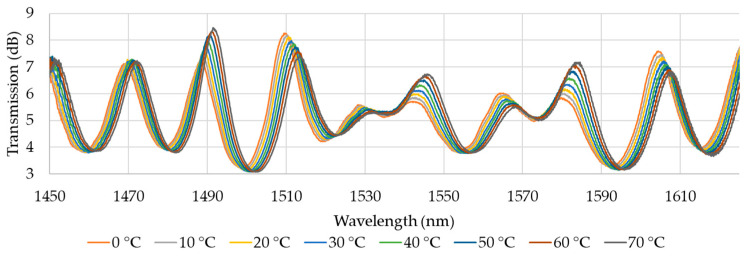
The transmission characteristics in S+C+L-bands as a function of temperature.

Sample analysis was carried out for the O-band and for S+C+L-bands. The results of the analysis are presented in Figure 6 and Figure 7, which show the transmission of the O-band (Figure 6) as a function of wavelength for different temperatures during the heating process and the transmission of the S+C+L-bands (Figure 7). The optical phase difference is the result of a change in the refractive index of the core and cladding modes, which causes a shift in the wavelength of the interference dips. This shift aligns with the predictions of Equation (4). Based on Equation (3) and the parameters from Table 2, the theoretical FSR can be calculated. As the reference, the transmission at 20 °C is established. For the O-band (Figure 6), the dips should appear every ~11.3 nm starting from 1312 nm. Presented data in Figure 6 confirm that dips are visible for expected wavelengths, and six dips can be distinguished with central wavelengths at 20 °C around 1292 nm, 1303 nm, 1312 nm, 1322 nm, 1327 nm, and 1338 nm. However, the dip at 1327 nm is not further investigated in this study. In the subsequent figure (Figure 7), eight dips are identified at central wavelengths of 20 °C: 1470 nm, 1489 nm, 1511 nm, 1529 nm, 1544 nm, 1565 nm, 1583 nm, and 1602 nm. The theoretical FSR calculated with Equation (3) is 18.6 nm. In the experiment, the average distance between transmission dips is 19.2 nm, which is convergent to the theory. The dip with a wavelength of 1529 nm is not included in the subsequent analyses due to its low amplitude. Additionally, the dip at λ = 1602 nm is excluded from the further presentation as it did not yield significant results. The average transmission level for the O-band is 3.5 dB, and for S+C+L-bands is 5.5 dB, which proves the high quality of developed MZI.

**Figure 8:**
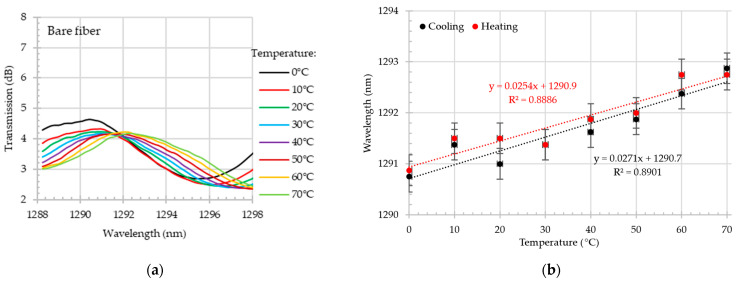
Bare MZI. Close-up on transmission spectrum in O-band for different temperatures in the range of 0–70 °C during the heating test (**a**); wavelength shift (with the error bars) as a function of temperature in O-band during the heating and cooling process (**b**).

**Figure 9:**
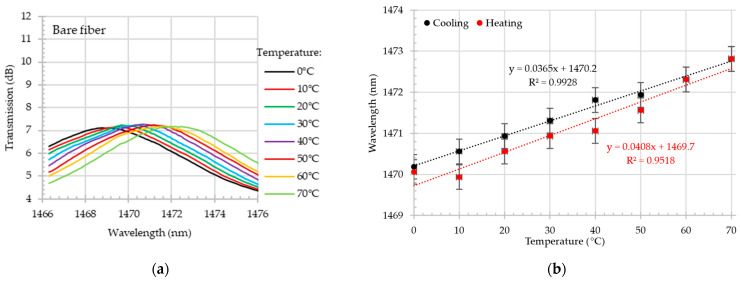
Bare MZI. Close-up on transmission spectrum in S+C+L-bands for different temperatures in the range of 0–70 °C during the heating test (**a**); wavelength shift (with the error bars) as a function of temperature in S+C+L-bands during the heating and cooling process (**b**).

As illustrated in Figure 8a, the close-up of the transmission dip shift in the O-band during the heating process reveals a wavelength shift from~1290.75 nm at 0 °C to 1292.85 nm at 70 °C. The wavelength shift for this dip during the cooling and heating process exceeds 25 pm/°C with the R^2^~0.89. In comparison, the sensitivity of the S-band dip, as depicted in Figure 9, exhibited a sensitivity that was approximately 40% higher. During the heating process, the dip shifted by 40.8 pm/°C. Additionally, the linearity of collected data is high—R^2^ > 0.95.

### 3.2. Gold-Coated Tapered Optical Fiber

**Figure 10:**
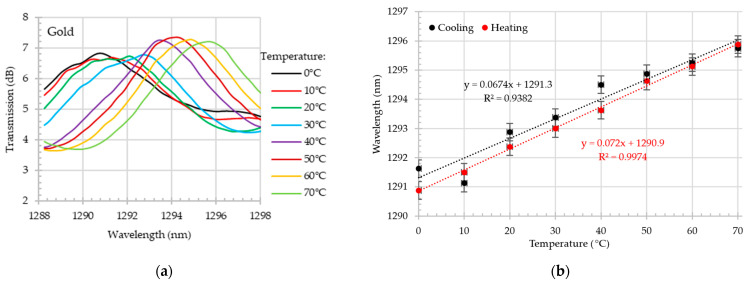
Gold-coated MZI. Close-up on transmission spectrum in O-band for different temperatures in the range of 0–70 °C during the heating test (**a**); wavelength shift (with the error bars) as a function of temperature in O-band during the heating and cooling process (**b**).

**Figure 11:**
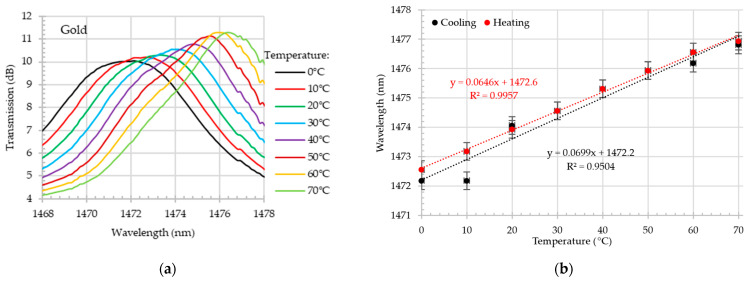
Gold-coated MZI. Close-up on transmission spectrum in S+C+L-bands for different temperatures in the range of 0–70 °C during the heating test (**a**); wavelength shift (with the error bars) as a function of temperature in S+C+L-bands during the heating and cooling process (**b**).

Much better results were obtained for gold-coated tapered optical fiber, as shown in Figure 10 and Figure 11. During the heating process, the first dip in the O-band reached 72 pm/°C, and during the cooling test, it reached 67.4 pm/°C. This is approximately three times more than the result for the sample without gold.

Additionally, the results collected in the O-band demonstrate the exceptional linearity of the developed sensor. Furthermore, the gold-coated tapered optical fiber demonstrated higher sensitivity in the S+C+L-bands compared to the bare tapered optical fiber. The wavelength shift of the gold-coated tapered optical fiber exceeded 64 pm/°C, reaching approximately 1.8 times the sensitivity of the uncoated tapered optical fiber while maintaining good linearity.

## 4. Discussion

Fiber optic sensors have several major advantages. They offer excellent sensitivity, remote operation over long distances, and are small and lightweight. In addition, they do not conduct electricity, which makes them immune to electromagnetic and radio frequency interference. For this reason, FBG or other types of fiber optic sensors are increasingly used to measure temperature in PV modules, making them commercially available. Since the sensitivity of a sensor is a key parameter, we proposed a high sensitivity type of sensor that can be easily integrated with PV modules, just like the FBG sensors. The minor discrepancies observed in the heating and cooling processes, as discussed in the preceding section, may be the result of temperature fluctuations within the thermal chamber. The climate chamber is designed to continuously adjust the internal temperature to the desired setting, thereby introducing potential discrepancies between the set temperature and the actual temperature within the chamber. The employment of a thermal chamber with enhanced temperature stabilization capabilities holds promise in resolving this issue.

**Figure 12:**
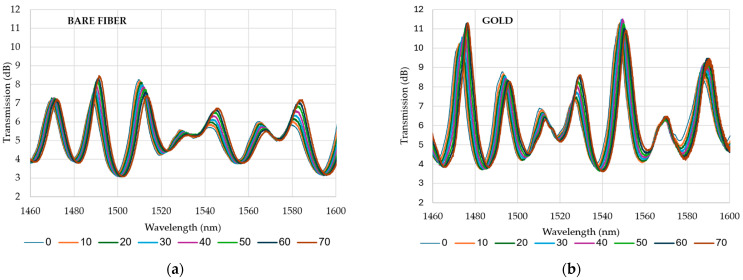
Transmission shift caused by temperature variation (**a**) for bare tapered optical fiber and (**b**) for gold-coated tapered optical fiber.

**Figure 13:**
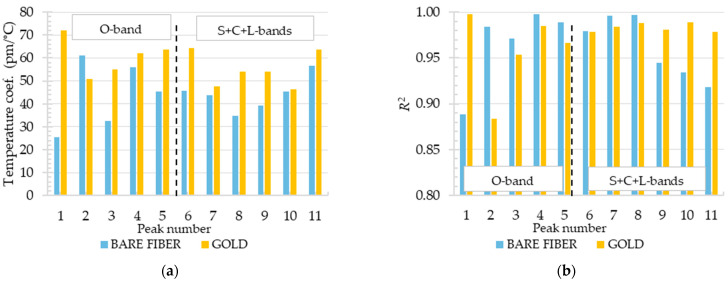
Transmission parameters. (**a**) Wavelength shifts of each transmission dip; (**b**) R^2^ coefficient of linear approximation.

**Figure 14:**
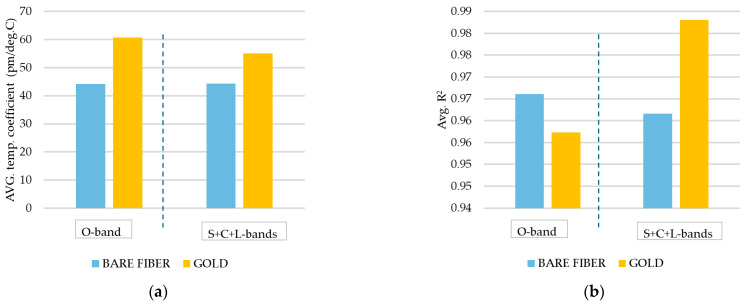
Transmission spectrum in O-band for different temperatures in the range of 0–70 °C during the cooling test (**a**); close-up on the shifted dips during the cooling test (**b**).

The deposition of a gold layer on a tapered optical fiber influences its transmission characteristics, as demonstrated in Figure 12. However, in the observed transmission spectra for all investigated bands, we did not observe a single significant SPR dip typical for the visible range of waves. On the other hand, our reference spectrum (shown in Figure 12a) already has dips caused by interferometry, and when a gold layer is applied, the amplitude of transmission dips is up to 80% higher. This may be the potential proof that SPR occurs, even if its efficiency is not that high. What is more interesting is that for the O-bands, gold deposition improved the average sensitivity more than for the S+C+L-bands (Figure 14a). This can be another evidence that SPR occurs and its efficiency is higher for the shorter waves than for S+C+L-bands. This evidence demonstrates that the gold layer enhances the leakage of light and improves the interference between the core mode and higher-order modes. Further investigation is required in order to study the behavior of the proposed sensor. Consequently, the average transmission attenuation is approximately 16% higher (~7 dB) for the gold-coated TOF compared to the bare TOF. However, the transmission attenuation of gold-coated TOF did not exceed 12 dB (Figure 12b), rendering it still highly useful. The sensitivity and coefficient of determination for each dip in the O-band and S+C+L-band were additionally investigated, as shown in Figure 13, and their average values are given in Figure 14. The results demonstrated that coating the TOF with gold improved the O-band sensitivity of the sensor by 40%. The average sensitivity of all dips exhibited an increase from 44 pm/°C to 60.7 pm/°C in O-band. A comparable sensitivity was observed for the bare TOF in S+C+L-bands, with a value of 44.2 pm/°C. Following the deposition of gold, the sensitivity increased to 55 pm/°C, which is 30% higher than the previous value. All types of sensors demonstrated satisfactory linearity, facilitating their calibration. In the O-band, the R2 of the second peak for gold-coated TOF exhibited a relatively low R^2^ value of 0.88. This influenced the average R^2^ value presented in Figure 14b.

As illustrated in Table 3, a comparative analysis of temperature sensors integrated into tapered optical fibers is presented, along with an assessment of their sensitivity. The presented data indicate that even the unmodified TOF demonstrates satisfactory high sensitivity, exhibiting an approximate sensitivity of 40 pm/°C. The incorporation of an additional layer of gold further enhances the potential of this construction, making it a promising subject for further research and development. In addition, our sensor offers one of the simplest constructions, which is a key feature from the point of view of fabrication costs and potential application on a larger scale.

## 5. Conclusions

In summary, an approach to measuring temperature with a compact Mach–Zehnder interferometer has been presented and demonstrated. Tapered optical fibers offer the advantages of a simple fabrication process and low production cost. The demonstrated sensitivity of the temperature sensors reached 72 pm/°C with R^2^ = 0.9974. The low average transmission loss of approximately 7 dB facilitates further optimization of the sensor, such as the fabrication of more abrupt tapers to enhance interference and increase sensitivity. This approach is compared with fiber Bragg gratings or F-P sensors reported to date, and it is demonstrated to have excellent sensitivity. The main advantages of the presented solution include a simple sensor design and high temperature sensitivity. The utilization of a standard telecom optical fiber with a standard telecom bandwidth of 1200–1600 nm is another key feature of our sensor and provides a long-distance application. On the other hand, the sensitivity can be further improved. In addition, infrared bandwidth does not support SPR as much as waves from the visible range and decreases its efficiency. In future work, there are several areas that we want to explore, such as the type and thickness of the layer used to coat the TOF and the influence of polarization on the obtained results. Additionally, we plan to conduct a numerical simulation in ANSYS Speos to calculate the field distribution in the sensor structure. The presented data show that a simple Mach–Zehnder interferometer structure based on only two tapers can have excellent parameters.

## Figures and Tables

**Table 1:** Parameters of the fusion splicer and sensor size.

*P*_arc_ [mW]/*t*_arc_ [s]	Waist Diameter *d* [µm]	Waist Length *l* [cm]	Space *l*_S_ [cm]
20/1.4	46 ± 1	0.74	2.4
30/1.4	33 ± 1	0.70	
20/1.6	42 ± 1	0.65	
30/1.6	26 ± 1	0.60	

**Table 2:** Calculation of FSR parameters.

Band	∆*n_eff_*	*L* [cm]	λ [nm]	Theoretical FSR [nm]	Experimental Avg. FSR [nm]
O-band	0.0047	3.2	1312	11.3	10.8
S+C+L-band	0.0042	3.2	1583	18.6	19.2

**Table 3:** Comparison to existing sensors.

Type of Sensor	Sensitivity	Ref.
Two tapers in series made on SMF	13.4 pm/°C	[62]
Tapered multicore fiber between two SMF	39.3 pm/°C	[63]
Tapered few modes fiber between two SMF	36.8 pm/°C	[64]
Tapered Multi-thin Multi-mode Fiber	75.04 pm/°C	[65]
MZI coated with polydimethylsiloxane	101 pm/°C	[66]
Two-mode fiber MZI	70 pm/°C	[67]
Multi-tapered PMF	78 pm/°C	[68]
MZI & FBG	35.18 pm/°C	[69]
Inline-MZI embedded point-shaped taper	73 pm/°C and 169 pm/°C	[70]
SMTMS	145.8 pm/°C	[71]
Gold-coated TOF on SMF	60 pm/°C (O-band) 55 pm/°C (S+C+L-bands)	This study

## Data Availability

The original contributions presented in this study are included in the article. Further inquiries can be directed to the corresponding author.

## Abbreviations

The following abbreviations are used in this manuscript:

TOF	Tapered Optical Fiber
FBG	Fiber Bragg Grating
PV	Photovoltaic
MZI	Mach–Zehnder interferometer
SPR	Surface Plasmon Resonance
SEM	Scanning Electron Microscope
EDS	Energy Dispersive X-ray Spectroscopy
